# Scaling a Community-Wide Campaign Intervention to Manage Hypertension and Weight Loss

**DOI:** 10.3389/fmed.2021.661353

**Published:** 2021-11-22

**Authors:** Belinda M. Reininger, Lisa A. Mitchell-Bennett, MinJae Lee, Paul G. Yeh, Amanda C. Davé, Soo Kyung Park, Tianlin Xu, Alma G. Ochoa-Del Toro

**Affiliations:** ^1^Health Promotion and Behavioral Sciences, Hispanic Health Research Center, University of Texas School of Public Health in Brownsville, Brownsville, TX, United States; ^2^Division of Biostatistics, Department of Population and Data Sciences, University of Texas Southwestern Medical Center, Dallas, TX, United States; ^3^Harold C. Simmons Comprehensive Cancer Center, University of Texas Southwestern Medical Center, Dallas, TX, United States; ^4^Department of Physician Assistant Studies, College of Health Professions, University of Texas Rio Grande Valley, Edinburg, TX, United States; ^5^Department of Biostatistics and Data Science, University of Texas Health Science Center, University of Texas School of Public Health at Houston, Houston, TX, United States

**Keywords:** US-Mexico border, community-wide campaign, evidence-based intervention, adults, hypertension, obesity, community health worker, RE-AIM framework

## Abstract

Public health impacts can be achieved when evidence-based interventions are implemented to those most in need. Too often implementation never or slowly occurs. The community-wide campaign intervention Tu Salud ¡Si Cuenta! has evidence of improving health outcomes related to chronic disease among low-income, Latinos. Using the RE-AIM Framework, this study examined if the scaled-up version of the intervention is associated with improvements in hypertension and obesity in 12 locations. Each element of the RE-AIM framework was examined. For “Effectiveness,” we examined outcomes overall and by implementing location. We used linear and logistic regression to assess if exposure in the intervention was associated with improvement in hypertension and weight loss. Participants were stratified into “low exposure” (2–3 outreach visits) vs. “high exposure” (4–5 outreach visits). Based on the RE-AIM Framework, the intervention “reached” its intended population of low-income Latinos, demonstrated “effectiveness” in improving hypertension and obesity, was “adopted” at a high level in all but one site, was “implemented” with fidelity to the intervention model with moderate success across locations, and showed high “maintenance” over time. For effectiveness specifically, we found that out of 5,019 participants, 2,508 (50%) had a baseline hypertensive blood pressure (BP) reading. Of the 2,508, 1,245 (49.9%) recovered to normal blood pressure or pre-hypertension stage by last follow-up. After adjusting for baseline BP and potential confounders in multivariable linear regression models, the high exposure group had significantly more reduction in systolic BP (adjusted mean difference in % change = −0.96; *p* = 0.002) and diastolic BP (adjusted mean difference in % change = −1.61; *p* < 0.0001) compared to the low exposure group. After controlling for baseline weight and other confounders, the high exposure group had significantly greater decrease in weight compared to the low exposure group (adjusted mean difference in % change = −1.28; *p* < 0.0001). Results from the multivariable logistic regression models indicated that compared to the low exposure group the high exposure group was more likely to achieve a clinically significant minimum 5% weight loss [adjusted odds ratio (OR) = 2.97; *p* < 0.0001). This study contributes evidence that a Community-Wide Campaign model holds promise for addressing hypertension and obesity among low-income Latinos.

## Introduction

### Hypertension in the U.S. Among Latinos

Hypertension increases the risk for cardiovascular disease including stroke, coronary artery disease, heart failure, atrial fibrillation, and peripheral vascular disease (1). In the U.S., hypertension and uncontrolled blood pressure are lower among Latino whites than other ethnic and racial groups (2). Additionally, hypertension prevalence was higher among non-Latino black (41.2%) than non-Latino white (28.0%), or Latino (25.9%) adults according to the National Health and Nutrition Examination Survey (NHANES) (2). This is not the case, however, on the U.S.-Mexico border in the Rio Grande Valley, Texas where 95% of the population is Latino (vast majority are of Mexican descent), and both men and women have a higher prevalence of hypertension than their counterparts across the nation, driven in part by local high rates of obesity (3). According to primary data from the Cameron County Hispanic Cohort, the burden of cardiovascular disease is highest among men in the region, with the prevalence of hypertension at 38.0% (3), as compared to the national overall prevalence of 26.1% for Latino men according to the NHANES (2).

### Obesity in the U.S. Among Latinos

Generally, for obese individuals, there is a dose-dependent response of health benefits with increased weight loss garnered. The American Diabetes Prevention Program trial established that a mean weight loss of 5% from baseline reduced the incidence of diabetes by >50% (4). Hamman et al. found that every kilogram of weight loss led to a 16% reduction in risk of progression to diabetes, and a 5% weight loss from baseline is associated with a 50% reduction in the incidence of type 2 diabetes (5). Additionally, the Look AHEAD trial demonstrated a strong linear correlation between decreases in hemoglobin A1c (an indicator of improved glycemic control) and increasing weight loss starting at 5% weight loss from baseline (6). Significant improvements in systolic and diastolic blood pressure, HDL cholesterol, and triglycerides were also seen beginning at this level of weight loss, again suggesting that there are cardiovascular health benefits tied to this clinically relevant criterion of weight loss (7).

### Community-Wide Campaign Models

Community-wide campaign (CWC) interventions have shown positive health outcomes in multiple settings and is, therefore, a recommended evidence-based strategy by the Centers for Disease Control and Prevention for certain outcomes (8). Improvement in physical activity (8), UV protection (9), folic acid usage (10, 11), child safety seat usage (12, 13) are some of the recent health outcomes that have been achieved using CWC interventions. The CWC intervention model involves multiple components including media, social support, health education, risk factor screening, environmental infrastructure change, and policy improvements (14, 15). Evaluation of the model has occurred in communities domestically such as Native Americans (16) or Mexican Americans (17–19), and internationally in Asia (20), Canada (21), and Finland (22, 23). Improvement in hypertension (24, 25) and obesity (26, 27) outcomes associated with CWC interventions have been studied in the past with mixed findings. Moreover, despite the overall effectiveness of CWC models, few studies have examined a scaled-up, *real-world* CWC program conducted across multiple communities (28).

### Research Aim

Our aim is to evaluate the public health impact of a multi-location CWC intervention on hypertension and obesity outcomes among low-income, Latino participants by examining the Reach, Effectiveness, Adoption, Implementation, and Maintenance of the program using the RE-AIM Framework (29). This CWC was scaled-up across 12 communities along the U.S. Mexico border between January 2014 and November 2019. Thought leaders have called for a greater focus on external validity in the evaluation of public health interventions to support more rapid uptake of effective public health interventions (30).

## Materials and Methods

### Setting and Method

The community-wide campaign, Tu Salud ¡Sí Cuenta! (TSSC, “Your Health Matters!” in English), has been implemented on the border in Texas with municipalities and county precincts to focus on the prevention of chronic diseases such as obesity, hypertension, and diabetes with programming that encourages fruit and vegetable intake and physical activity. This evidence-based CWC incorporates mass media messaging, social support, health education, risk factor screening, as well as environmental infrastructure change and policies to achieve health outcomes. TSSC is culturally tailored to a predominantly Latino population, employing local community health workers (CHWs), and certified instructors within each of the partner communities who lead the efforts of social support, risk factor screening, exercise groups, and healthy cooking classes. These lay health promoters are the voices of their communities, acting as liaisons between city officials and residents to advocate for local needs. CHWs are trained to collect information from individuals ranging from demographic factors to current levels of physical activity and fruit and vegetable consumption as well as blood pressure and body mass index (BMI) measurements. Given healthcare provider shortages and systemic issues of healthcare inaccessibility for the local population, CHWs play a critical liaison role, tasked with delivering health education using motivational interviewing strategies to promote healthy lifestyle changes and following participants' progress. Past research on this initiative has demonstrated improved health outcomes among participants, including meeting physical activity guidelines, reducing sedentary behavior, and increasing consumption of fruits and vegetables (17, 18). One study examined a six session home visit curriculum delivered by the CHWs found significant improvements in physical activity at 6 months to those exposed to at least three or more sessions (19).

All municipal and precinct locations involved in this study are located along the U.S.-Mexico border with Texas in the Rio Grande Valley (RGV). The region is young (mean age of 45 years) (31) and rapidly growing (32), with a 2010 population of over 1.3 million, of which over 93% are Latino (3, 32). This area has over one third of the population living in poverty (33, 34), with its per capita income half the American average and low education levels (32). As is common in persistent poverty regions, health disparities abound (33). For example, over 84% of adults are obese or overweight (3). Additionally, nearly 67% of the adult population lacks health insurance (31).

#### Implementing Locations

**Figure 1:**
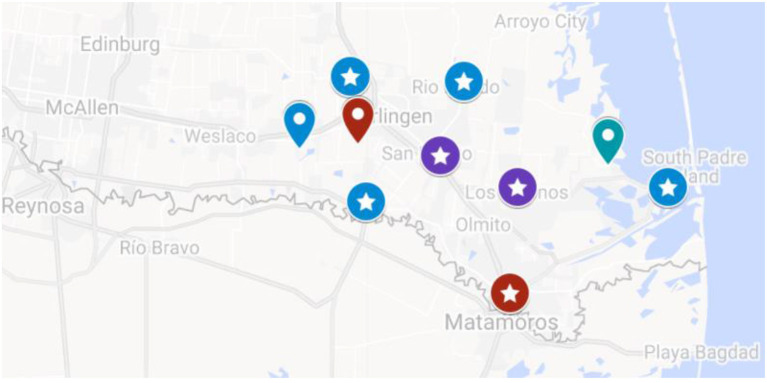
Map of Participating Implementing Locations of the TSSC Program. Google map showing the location of the 12 municipalities in the TSSC study. Red labels are “Cities” with population sizes ranging from 75,000 to 200,000. Purple labels are “Towns” with a population size from 5,000 to 70,000. Blue labels are “Rural” areas with a population of <5,000. Labels with stars indicate the municipalities with sufficient sample size (*n* ≥ 35) that were included in the municipal-level analysis.

The TSSC program has been disseminated and implemented in 12 locations. There were two county precincts with a population of 190,000 each, and 10 municipalities including two major urban areas with a population of over 75,000 each (“City”), two small towns that ranged in population from 5,000 to 70,000 (“Town”), and six rural areas with populations of <5,000 (“Rural”) (32). A map of the locations is provided in Figure 1.

### Measurement

#### RE-AIM Framework

This public health evaluation framework examines five factors to conceptualize the impact of an intervention in a population. Reach, effectiveness, adoption, implementation, and maintenance are separate dimensions that span the individual and organizational influences of an intervention and thereby provide evaluative insight into interventions that may work in real-world environments (35, 36).

#### Socio-Demographic Characteristics

Sociodemographic characteristics collected from the sample at baseline included: sex (male/female), age (respondent's date of birth), Latino (yes/no), health insurance (insured with government or private health insurance or uninsured), and income [above or below the federal poverty level (37)]. Income level was based on two questions regarding household size and yearly household income. These variables were used in multivariable regression models of effectiveness as well as to discuss the reach of the program in terms of the intended priority population. For the analysis, we only included those who identified as Latino given the low sample size that identified as non-Latino.

#### Blood Pressure

Blood pressure was measured by the same CHW at baseline and follow-up for each participant. The blood pressure was measured according to protocol, with the participant seated and blood pressure taken twice with the right arm using an electronic blood pressure machine, calibrated yearly, and assigned to the same CHW so that the same machine was used to ensure consistent readings. The blood pressure was recorded by the CHW in an online data management system.

**Figure 2:**
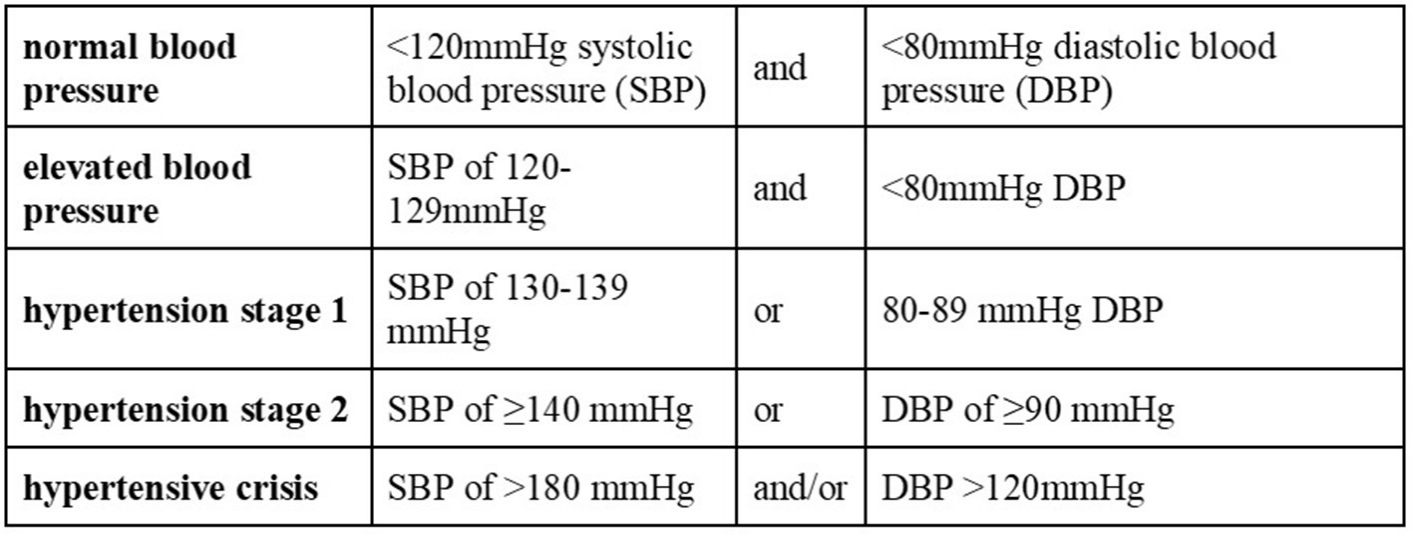
Blood pressure categories used for analysis of the study's participant based on the American College of Cardiology (ACC) and American Heart Association's (AHA) 2017 blood pressure guidelines (38).

Blood pressure was categorized per the American College of Cardiology (ACC) and American Heart Association's (AHA) 2017 blood pressure guidelines (Figure 2) (38).

The American College of Cardiology/American Heart Association (ACC/AHA) 2017 guidelines on hypertension indicates that 65 years of age is the recommended cutoff for standard hypertension categories (e.g., prehypertension, hypertension stage I/II) with the criteria for “normal” blood pressure being liberalized to 130/90 from 120/80 (38). This is to account for those over 65 years of age naturally having intrinsically increased systolic blood pressure and declining diastolic blood pressure due to age-related physiological changes (39). In tandem with the ACC/AHA guidelines recognizing the unique blood pressure profiles and adverse cardiovascular events specific to those over 65 years of age (38, 40), we have limited our study to individuals 65 years of age or younger.

Another exclusion criterion from the analysis are patients with a measured pulse pressure (PP, measured as one's systolic–diastolic blood pressure) >100 mmHg (also known as isolated systolic hypertension). A PP > 100 is associated with left ventricular hypertrophy (41), higher cardiovascular-related adverse events and mortality rate (42), and impacts their response to antihypertensive measures including behavioral lifestyle modifications and/or medications (43). There is also a clinical issue with anti-hypertensive therapy in individuals with a wide pulse pressure (especially if diastolic blood pressure is <55 mmHg) because we risk lowering the diastolic blood pressure excessively and inadvertently increasing coronary risk due to diastolic hypotension (44). The exclusion of those with a PP > 100 or those individuals with large fluctuations in systolic or diastolic blood pressure (>100 mmHg change or >100% change from baseline) is to eliminate the confounding effects of isolated systolic hypertension and diastolic hypotension on this intervention that is seeking to ascertain the reduction of overall blood pressure. Indeed, such individuals experienced a drop of blood pressure that is unsafe clinically and may suggest that these individuals were not medically appropriate for a CHW-based intervention and require higher levels of medical care for their hypertension (44).

#### Body Mass Index and Weight

Each participant had an assigned CHW who used an electronic scale to measure the weight of the participant and a standard measuring tape to measure the patient's height (in meters). The scales were calibrated every 6 months and are able to measure weight up to 400 pounds. BMI was then calculated dividing the kg in mass of a participant with the meters squared of the height of the participant. This was done for each participant at each CHW visit. The BMI was used to classify individuals into normal weight (BMI < 25), overweight (BMI 25–30), and obese (BMI > 30) (45). Additionally, participants were monitored for achieving 5% weight loss, which has been a threshold generally accepted to indicate clinically meaningful weight loss (46).

#### Participant Assignment to Locations

CHWs enrolled participants and documented enrollment by geographic location. The location corresponded to the community in which participants received TSSC services and participated in program activities. Most participants were seen by the CHW assigned to serve within the boundaries of the precinct or municipality where they reside, with some exceptions. The University employed three CHWs who served across geographic locations so the participants they reached were grouped by their primary residence.

### Statistical Analysis

Based on the RE-AIM Framework (29, 35, 47–49) for evaluating public health interventions, the following analysis approach was conducted.

#### Reach

Reach was ascertained by calculating the percentage of unique TSSC program participants who agreed to have TSSC program CHW follow-up visits of the total population living in the municipalities and precincts during the study timeframe. Special consideration was also made in assessing the ethnicity and poverty status of the enrolled participants as compared to the general population of the partnering municipalities and precincts. The total population of the municipalities/precincts (“locations”) was based on the 2018 American Community Survey (34).

#### Effectiveness

**Figure 3:**
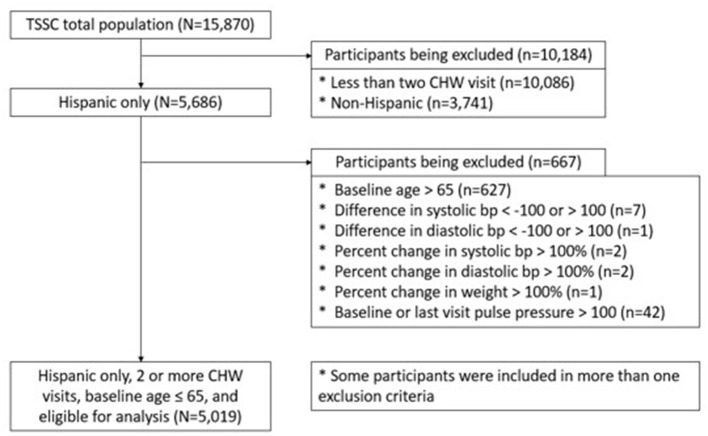
Flowchart for the selection of the study's sample.

Among the total 15,870 TSSC study participants entered into the program database, we included 5,019 participants in our statistical analyses after excluding *n* = 10,086 subjects who had <2 CHW visits, *n* = 3,741 non-Latino participants, and *n* = 667 subjects based on the exclusion criteria for this study including age of participant and inaccurate/untenable values of BP or weight changes (Figure 3). We classified participants into two groups based on total number of visits: (1) high exposure group (*n* = 904, 18.01%), those who made 4 or 5 CHW visits, and (2) low exposure group (*n* = 4,115, 81.99%), those with 2 or 3 CHW visits. We assessed whether the TSSC program contributed to improvement of participants' blood pressure (BP) level or weight/BMI. Notably, the terminology of “improvement” of these outcome variables as used in this study signifies decreases in SBP, DBP, weight, and BMI. Specifically, we calculated the percent changes in each outcome variable from the baseline to the last visit and compared these changes between high and low exposure groups.

We first conducted univariable comparisons of baseline characteristics of participants between two study groups (“low exposure” vs. “high exposure”) using Students' *t*-test (or its non-parametric counterpart Wilcoxon test, if appropriate) for continuous variables and Chi-square test for categorical variables. We then compared the percent changes in systolic and diastolic BP levels or weight from baseline to the last visit between high and low exposure groups using univariable and multivariable linear regression models with adjustment of participants' baseline value of each outcome and duration of participation. We also conducted subgroup analyses using univariable and multivariable logistic regression models for the binary outcome variables of interest as follows. We looked at 3,519 participants (70.11%) who had hypertensive (*n* = 2,508) or elevated BP (*n* = 1,011) at baseline, and compared the number of participants who recovered to normal blood pressure after participating in the TSSC program between high and low exposure groups. In addition, we assessed whether the program helped participants achieve a minimum 5% weight loss among 3,272 (65.19%) who had weight loss. Furthermore, we investigated 3,075 (61.27%) participants who were obese at baseline, and assessed the number of those who improved to overweight or normal weight after the program between high and low exposure groups. In addition to these aforementioned main analyses, we investigated each of outcomes by location that was implemented with fidelity. We used the same models of linear regression and logistic regression analyses for the location-specific analysis as the analysis on the overall population. Potential confounding variables were selected a priori based on our previous studies, and examined and incorporated during development of the final multivariable models for each outcome. SAS 9.4 (SAS Institute Inc., Cary NC) was used to perform all statistical analyses and statistical significance was assumed at the 0.05 level.

#### Adoption

Census data (34) was used to identify the total number of municipalities/precincts in the two-county region. We examined historical records and contracts for the number of municipalities/precincts offered and the number that implemented the program between January 2014 and November 2019.

#### Implementation

Fidelity for this study was based on evidence of at least 35 participants with documented CHW home visits in both the high exposure (4–5 visits) and low exposure (2–3 visits) categories to be included in the location-specific effectiveness analysis. In regards to CHW home visits, past research on CHW home visits associated with this initiative and based on a specifically delivered curriculum to guide the visits showed that exposure to 3 or more monthly visits was associated with significant improvements in physical activity at 6 months, but at 12 months significance was not retained (19). Therefore, the program defined fidelity as enough of a sample size for stable regression analysis with 4 or more monthly visits to enhance long-term retention of outcomes. The data for the number of visits were logged by the CHWs into an electronic database and participant-level data that were captured in a case management database documenting services delivered and anthropometric data overtime.

#### Maintenance

This dimension was defined as the number of locations that adopted after January 2014 and had an active program until November 2019.

## Results

### Reach

The total population of the 12 locations in which the TSSC program was implemented was 718,647 (34). During the study period, 15,870 participants agreed to enrollment and a follow-up visit where health education was provided by a CHW. These participants represent 2.21% of the total population. Of the participants who provided ethnicity data, 93.25% identified as Latino, slightly more than the Latino population living in these 12 locations based on the latest Census data (32, 34). Among participants who provided both household size and income information, 81.18% were below the Federal Poverty Line (37). Based on the census data for these locations, approximately 30.9% of the population live at or below the poverty level (34). The intervention fully reached the priority population, with 81.91% of the participants who provided ethnicity and income data were found to be Latinos with low income (37).

### Effectiveness: Baseline Characteristics

Among 15,870 total TSSC participants, 10,184 participants who either had only one CHW visit (*n* = 10,086, 63.6%; as no comparison follow-up measure of blood pressure and weight from baseline could be examined) or did not specifically identify as Latino (*n* = 3,741, 23.6%) were excluded from the analysis, as seen in Figure 3. Additional exclusion criteria including age of participant and inaccurate/untenable values of BP or weight changes led to a final sample of 5,019 (31.6%) being included in the final analysis. These individuals were classified into “low exposure” (*n* = 4,115) and “high exposure” (*n* = 904) groups for analytical comparisons.

Baseline demographic characteristics by study group are presented in Table 1. The majority of the study population was female participants (79.10%). Participants in the high exposure group were slightly older (44.99 vs. 43.83 years; *p* = 0.0060) and less likely to be below the federal poverty level (FPL) (74.94 vs. 84.19%; *p* < 0.0001) than the low exposure group. Also, the high exposure group was more likely to have insurance coverage compared to the low exposure group (44.30 vs. 35.55%; *p* < 0.0001). Baseline weight, SBP and DBP were slightly higher in the high exposure group compared to those in the low exposure group. We found more participants who had hypertensive BP at baseline in the high exposure group compared to the low exposure group. The low exposure group had more participants who had normal BP or normal BMI at baseline (Table 1). Median length of follow-up was 3.23 months [IQR = (1.33, 5.93)] {2.33 months [IQR = (1.10, 4.90)] for the low exposure group; 6.53 months [IQR = (4.90, 9.07)] for the high exposure group}. Univariable comparisons of changes in each outcome between exposure groups were also shown in Table 1.

**Table 1:** Baseline characteristics and descriptive changes of outcomes (*N* = 5,019).

**Variable**	**All** **(***N*** = 5,019)**	**Low exposure** **(***N*** = 4,115)**	**High exposure** **(***N*** = 904)**	***P*** **-value^a^**
Age, years, mean (SD)	44.04 (11.42)	43.83 (11.49)	44.99 (11.05)	0.0060
Female, *N* (%)	3,970 (79.10%)	3,260 (79.22%)	710 (78.54%)	0.6477
Have insurance, *N* (%)	1,863 (37.13%)	1,463 (35.55%)	400 (44.30%)	< .0001
Number of program strategies received^b^, median (IQR), [min, max]	2 (1, 2) [1, 8]	2 (1, 2) [1, 7]	2 (1, 2) [1, 8]	0.0095
Below federal poverty level^*^, *N* (%)	3,561 (82.49%)	2,966 (84.19%)	595 (74.94%)	< .0001
Self-reported diabetes at baseline, *N* (%)	704 (14.03%)	580 (14.09%)	124 (13.72%)	0.7670
Hypertensive BP at baseline, *N* (%)	2,508 (49.97%)	2,017 (49.02%)	491 (54.31%)	0.0039
Baseline SBP, mmHg, mean (SD)	127.39 (15.60)	126.50 (14.87)	131.40 (18.07)	<0.0001
Baseline DBP, mmHg, mean (SD)	76.30 (10.81)	76.16 (10.52)	76.93 (12.01)	0.0750
Baseline BP categories, *N* (%)				<0.0001
Normal	1,500 (29.89%)	1,261 (30.64%)	239 (26.44%)	
Elevated	1,011 (20.14%)	837 (20.34%)	174 (19.25%)	
High BP (Hypertension) Stage 1	1,544 (30.76%)	1,330 (32.32%)	214 (23.67%)	
High BP (Hypertension) Stage 2	935 (18.63%)	668 (16.23%)	267 (29.54%)	
Hypertensive crisis	29 (0.58%)	19 (0.46%)	10 (1.11%)	
Baseline weight, lb., mean (SD)	183.43 (39.32)	182.50 (38.39)	187.7 (43.10)	0.0009
Baseline BMI, kg/m^2^, N (%)				0.0049
Normal (<25)	259 (5.16%)	231 (5.61%)	28 (3.10%)	
Overweight (25–29.9)	1,685 (33.57%)	1,388 (33.73%)	297 (32.85%)	
Obese (>30)	3,075 (61.27%)	2,496 (60.66%)	579 (64.05%)	
Changes based on BP levels or weight				
Mean % Change in SBP baseline to last visit, mean (SD)	−3.08 (9.01)	−2.64 (8.53)	−5.12 (10.73)	<0.0001
Mean % Change in DBP baseline to last visit, mean (SD)	−2.36 (12.7)	−2.11 (12.22)	−3.49 (14.87)	0.0096
Mean % Change in weight baseline to last visit, mean (SD)	−1.41 (4.38)	−1.09 (4.17)	−2.84 (4.96)	<0.0001
Mean % weight loss (among *n* = 3272 who have weight loss), mean (SD)	−3.05 (3.81)	−2.61 (3.59)	−4.83 (4.11)	<0.0001
% participants who had weight loss, *N* (%) (#missing = 20)	3,272 (65.45%)	2,619 (63.92%)	653 (72.39%)	<0.0001
Changes based on hypertension or BMI categories^**^				
**Among the participants who had “HTN” stage at baseline (#missing** **=** **14)‡**	**All** (*N* = 2,508)	**Low exposure** (*n* = 2,004)	**High exposure** (*n* = 490)	***P*** **-value** ^a^
#changing from HTN Stage to Pre-HTN stage (Normal/Elevated BP), *N* (%)	1,245 (49.92%)	1,007 (50.25%)	238 (48.57%)	0.5054
**Among the participants who had “HTN” or “elevated” Stage at baseline (#missing = 15)#**	**All** (*N* = 3,519)	**Low exposure** (*n* = 2,840)	**High exposure** (*n* = 664)	***P*** **-value** ^a^
#changing from HTN/elevated Stage to normal BP stage, N (%)	970 (27.68%)	778 (27.39%)	192 (28.92%)	0.4302
**Among the participants who had any weight loss**	**All** (*N* = 3,272)	**Low exposure** (*n* = 2,619)	**High exposure** (*n* = 653)	***P*** **-value** ^a^
#Weight loss > 5% from baseline to last visit, N (%)	519 (15.86%)	276 (10.54%)	243 (37.21%)	<0.0001
**Among the participants who were obese at baseline (#missing = 13)**	**All** (*N* = 3,075)	**Low exposure** (*n* = 2,483)	**High exposure** (*n* = 579)	***P*** **-value** ^a^
#changing from obese to overweight/normal (pre-obese) BMI category, N (%)	238 (7.77%)	158 (6.36%)	80 (13.82%)	<0.0001

a *T-test or its non-parametric equivalent (i.e., Wilcoxon rank sum test) for continuous variables and Chi-square test for categorical variables were used*.

b *Number of program strategies received per participant has a range from 1 to 8 and includes newsletter, exercise classes, weight loss support groups, health education programming, motivational text messaging, and risk factor screening*.

* *n = 702 (14%) were missing*.

** *n = 13~15 were missing due to some records missing at the last visit*.

‡ *Hypertension stage 1 (n = 1,544) + Hypertension stage 2 (n = 935) + Hypertensive Crisis (n = 29)*.

# *Elevated (n = 1,011) + Hypertension stage 1 (n = 1,544) + Hypertension stage 2 (n = 935) + Hypertensive Crisis (n = 29)*.

*All participants received at least 2 community health worker home visits as part of the inclusion criteria for this study*.

### Effectiveness Outcomes

We found that the TSSC program significantly improved participants' blood pressure levels and weight in both univariable and multivariable models. Mean percent changes in SBP, DBP, and weight for the high exposure group were significantly lower (i.e., more reduction) than those for the low exposure group. The high exposure group had more participants who had weight loss compared to the low exposure group (Table 1). Unadjusted results from univariable models remained very similar to the adjusted results of multivariable models. As shown in Table 2, after adjusting for potential confounders such as age, gender, insurance, components received, income below FPL, and baseline comorbidities such as hypertension, diabetes, and obesity, the high exposure group had more reduction in systolic and diastolic BP compared to the low exposure group (adjusted mean % change from baseline to last visit: −3.41 vs. −2.45% (SBP) and −3.52 vs. −1.91% (DBP); adjusted mean difference between high vs. low exposure: −0.96% (*p* = 0.0021) and −1.61% (*p* < 0.0001) for SBP and DBP, respectively). In addition, based on the multivariable model, the high exposure group had greater weight loss compared to the low exposure group [adjusted mean % change from baseline to last visit: −2.32 vs. −1.04%; adjusted mean difference between high vs. low exposure: −1.28%; (*p* < 0.0001)]. Females were significantly associated with more reduction in both BP levels and weight. A higher number of program strategies received was significantly associated with more reduction in weight (*p* = 0.03). Insured participants had less decrease in SBP (*p* = 0.06) and DBP (*p* = 0.007) compared to uninsured participants. Being below FPL was associated with less decrease in SBP (*p* = 0.26), DBP (*p* = 0.04), and weight (*p* = 0.21).

**Table 2:** TSSC program effect on change in systolic and diastolic BP from baseline to last visit based on linear regression analysis (*N* = 5,019).

	**Percent change in systolic BP from baseline to the last CHW visit**		**Percent change in diastolic BP from baseline to the last CHW visit**		**Percent change in weight from baseline to the last CHW visit**	
**Variable**	**Adjusted mean difference in %change**	***P*** **-value**	**Adjusted mean difference in %change**	***P*** **-value**	**Adjusted mean difference in %change**	***P*** **-value**
Exposure high vs. low	**−0.96 (−1.57, −0.35)**	**0.0021**	**−1.61 (−2.42, −0.81)**	**< .0001**	**−1.28 (−1.64, −0.92)**	**<0.0001**
Estimated Mean change (95% CI) from baseline to the last CHW visit in each group	***Low:** **−2.45 (−2.87, −2.03)*** ***High:** **−3.41 (−4.05, −2.77)***		***Low:** **−1.91 (−2.46, −1.35)*** ***High:** **−3.52 (−4.36, −2.68)***		***Low:** **−1.04 (−1.29, −0.79)*** ***High:** **−2.32 (−2.70, −1.94)***	
#program strategies received	0.17 (−0.05, 0.40)	0.1349	0.13 (−0.17, 0.43)	0.4093	−0.15 (−0.28, −0.01)	0.0336
Age, year	0.03 (0.005, 0.05)	0.0155	−0.01 (−0.03, 0.02)	0.5893	−0.01 (−0.02, 0.01)	0.3494
Sex female vs. male	−1.53 (−2.10, −0.97)	<0.0001	−1.46 (−2.20, −0.71)	0.0001	−0.50 (−0.84, −0.16)	0.0039
Have insurance yes vs. no	0.46 (−0.01, 0.92)	0.0560	0.86 (0.24, 1.48)	0.0066	−0.07 (−0.35, 0.21)	0.6411
Poverty status below vs. above FPL	0.34 (−0.25, 0.93)	0.2559	0.83 (0.05, 1.60)	0.0370	0.22 (−0.13, 0.58)	0.2096
Baseline diabetes Yes vs. no	0.72 (0.09, 1.34)	0.0246	0.50 (−0.33, 1.33)	0.2337	0.20 (−0.18, 0.57)	0.3018
Baseline obesity Yes vs. no	1.05 (0.61, 1.49)	<0.0001	1.18 (0.59, 1.77)	<0.0001	−0.15 (−0.42, 0.11)	0.2573

*The bold values indicate the overall MAIN effect of the program on systolic BP, diastolic BP, and weight change, whereas the italicized format indicates the differential program effect to subgroups of participants classified in the “low” program exposure and the “high” program exposure groups. The italicized data thus presents specific group data compared to the overall main effect data in bold*.

### Effectiveness Outcomes Across Municipalities on Blood Pressure

We also looked at 3,519 participants (70.11%) who had hypertensive or elevated BP at baseline, and compared the number of participants who recovered to normal blood pressure after receiving the intervention between high and low exposure groups using logistic regression models. Out of the 3,519 participants, 970 (27.68%) recovered to normal blood pressure at their last visit, and we found that the high exposure group had slightly more participants who recovered from hypertension or elevated BP compared to low exposure group (28.92 vs. 27.39%, *p* = 0.4302, Table 1). However, the results based on multivariable logistic regression model (Table 3), after adjusting for the potential confounding factors as well as participants' baseline data and duration of participation, showed that the low exposure group was more likely to recover to normal BP level from hypertensive or elevated BP than the high exposure group [adjusted odds ratio (OR) = 0.92; 95% CI = (0.73, 1.15)], but this finding was not statistically significant (*p* = 0.4472). Females were more likely to recover to normal BP level, while older age, diabetes, obesity, having insurance, being above FPL at baseline were significantly associated with lower odds of recovering to normal BP among those who had hypertensive or elevated BP at baseline (Table 3). A total of 1,245 participants (49.92%) were recovered to a pre-hypertension stage (i.e., normal/elevated BP) among those with hypertension at baseline, but there was no significant differences found between the high (49%) and low exposure (50%) groups (Table 1).

**Table 3:** TSSC program effect on changing from HTN/Elevated Stage to Normal BP stage based on logistic regression analysis (*n* = 3,519).

	**Unadjusted**		**Adjusted**	
**Variable**	**Odds Ratio (95% CI)**	***P*** **-value**	**Odds Ratio (95% CI)**	***P*** **-value**
Exposure high vs. low	**0.93 (0.76, 1.14)**	**0.4971**	**0.92 (0.73, 1.15)**	**0.4472**
# program strategies received			1.05 (0.96, 1.15)	0.2743
Age, year			0.98 (0.97, 0.99)	< .0001
Sex female vs. male			2.36 (1.90, 2.94)	< .0001
Have insurance yes vs. no			0.78 (0.65, 0.93)	0.0049
Poverty status below vs. above FPL			0.80 (0.64, 0.99)	0.0471
Baseline diabetes yes vs. no			0.78 (0.61, 0.99)	0.0379
Baseline obesity yes vs. no			0.67 (0.57, 0.80)	< .0001

*The meaning of the bold values provided in the unadjusted and adjusted program effect on changing from abnormal to normal BP*.

### Effectiveness Across Municipalities on Weight

Further, we assessed whether the program helped participants achieve a clinically significant minimum 5% weight loss by conducting logistic regression models. Among 3,272 participants who had weight loss, 519 (15.86%) participants lost more than 5% of their weight, and we found that high exposure group was more likely to achieve a minimum 5% weight loss compared to the low exposure group (37.21 vs. 10.54%, *p* < 0.0001, Table 1). This difference was also found statistically significant based on the multivariable logistic regression [adjusted OR = 2.97; 95% CI = (2.33, 3.80); *p* < 0.0001] (Table 4). The participants who were obese at baseline had significantly higher odds of losing more than 5% of weight over the participation in the program. Also, the greater the number of program strategies received, the higher the odds of losing more than 5% of weight among the participants who had weight loss (Table 4).

**Table 4:** TSSC program effect on weight loss > 5% among the participants who had weight loss based on logistic regression analysis (*n* = 3,272).

	**Unadjusted**		**Adjusted**	
**Variable**	**Odds Ratio (95% CI)**	***P*** **-value**	**Odds Ratio (95% CI)**	***P*** **-value**
Exposure high vs. low	**3.13 (2.50, 3.91)**	**<0.0001**	**2.97 (2.33, 3.80)**	**<0.0001**
#program strategies received			1.13 (1.00, 1.27)	0.0427
Age, year			0.99 (0.98, 0.99)	0.0077
Sex female vs. male			1.10 (0.82, 1.47)	0.5248
Have insurance yes vs. no			1.05 (0.83, 1.32)	0.6955
Poverty status below vs. above FPL			0.79 (0.60, 1.04)	0.0959
Baseline diabetes yes vs. no			1.05 (0.76, 1.44)	0.7901
Baseline hypertension yes vs. no			1.17 (0.93, 1.47)	0.1870
Baseline obesity yes vs. no			1.29 (1.01, 1.63)	0.0381

*The meaning of the bold values provided in the unadjusted and adjusted program effect on getting 5% weight loss*.

Lastly, we investigated 3,075 (61.27%) participants who were obese at baseline, and assessed the number of participants who improved to either overweight or normal weight after the program between high and low exposure group using logistic regression models. Out of 3,075 participants, 238 (7.77%) had improved to either overweight or normal weight, with the high exposure group more likely to obtain this improvement than the low exposure group (13.82 vs. 6.36%, *p* < 0.0001, Table 1). This difference was also confirmed to be statistically significant based on the multivariable logistic regression model [adjusted OR = 1.93; 95% CI = (1.37, 2.71); *p* = 0.0002] (Table 5). The insured participants at baseline were significantly more likely to improve to either overweight or normal weight, while hypertension at baseline was significantly associated with lower odds of changing to overweight or normal weight among those who were obese at baseline (Table 5). There were a relatively small number of the participants who were obese or overweight at baseline who achieved normal weight (*n* = 103, 2.17%) by posttest, and no difference was found between high and low exposure groups (Table 1).

**Table 5:** TSSC program effect on changing from Obese to Overweight/Normal (pre-obese) BMI category based on logistic regression analysis (*n* = 3,075).

	**Unadjusted**		**Adjusted**	
**Variable**	**Odds Ratio (95% CI)**	***P*** **-value**	**Odds Ratio (95% CI)**	***P*** **-value**
Exposure high vs. low	**1.79 (1.31, 2.46)**	**0.0003**	**1.93 (1.37, 2.71)**	**0.0002**
#program strategies received			1.11 (0.96, 1.27)	0.1540
Age, year			1.01 (1.00, 1.03)	0.0565
Sex female vs. male			0.97 (0.66, 1.44)	0.8902
Have insurance yes vs. no			1.37 (1.02, 1.85)	0.0382
Poverty status below vs. above FPL			0.92 (0.64, 1.32)	0.6489
Baseline diabetes yes vs. no			0.88 (0.59, 1.30)	0.5090
Baseline hypertension yes vs. no			0.56 (0.42, 0.76)	0.0001

*The meaning of the bold values provided in the unadjusted and adjusted program effect on changing from obese BMI to overweight/normal BMI*.

### Effectiveness by Location on Blood Pressure and Weight

We conducted location-specific analyses (Table 6) for seven different locations who reported sufficient participation. The analysis from 1 City, 2 Towns, and 4 Rural areas used the same regression models that we used for the overall population. The proportion of their sample that fell in the high exposure group varied greatly across locations and was documented as 31.7% of *n* = 1,146 (City A); 7.1% of *n* = 436 (Town A); 7.8% of *n* = 677 (Town B); 56% of *n* = 597 (Rural A); 13% of *n* = 432 (Rural B); 8.3% of *n* = 397 (Rural C); 6.7% of *n* = 538 (Rural D). Based on multivariable regression models, we found that the TSSC program improved participants' SBP and DBP levels and weight in most of the locations. Both the high and low exposure groups had improved participants' SBP levels in all locations except Rural C. The high exposure group had significantly more reduction in SBP levels in City A and Rural A. Though not statistically significant, we also found the high exposure group had more improvement (decrease) of SBP in Town A, B, and Rural B and D.

**Table 6:** TSSC program effect on change in BP level and weight/BMI by location based on multivariable regression analysis.

**Mean difference in % change of SBP**							
	**City A** **(***n*** = 1,146)**	**Town A** **(***n*** = 436)**	**Town B** **(***n*** = 677)**	**Rural A** **(***n*** = 597)**	**Rural B** **(***n*** = 432)**	**Rural C** **(***n*** = 397)**	**Rural D** **(***n*** = 538)**
Adjusted mean difference in change and 95% confidence interval with *p*-value							
Exposure high vs. low	−2.19 (−3.50, −0.88); *p* = 0.0011	−1.23 (−4.19, 1.73); *p* = 0.4143	−0.34 (−1.80, 1.13); *p* = 0.6537	−1.71 (−3.26, −0.17); *p* = 0.0301	−1.08 (−2.87, 0.72); *p* = 0.2382	2.55 (−0.75, 5.84); *p* = 0.1288	−0.41 (−2.32, 1.51); *p* = 0.6766
Estimated Mean change (95% CI) from baseline to the last CHW visit in each group	Low: −2.03 (−3.10, −0.95) High: −4.21 (−5.60, −2.83)	Low: −1.85 (−3.44, −0.25) High: −3.08 (−6.32, 0.17)	Low: −1.85 (−2.74, −0.97) High: −2.19 (−3.78, −0.60)	Low: −2.23 (−3.66, −0.81) High: −3.95 (−5.27, −2.63)	Low: −5.10 (−6.13, −4.10) High: −6.17 (−8.02, −4.33)	Low: −0.56 (−2.87, 1.74) High: 1.99 (−2.26, 6.24)	Low: −1.26 (−3.00, 0.47) High: −1.67 (−3.97, 0.63)
**Mean difference in % change of DBP**							
Exposure high vs. low	−2.33 (−4.00, −0.67); *p* = 0.0060	−3.17 (−6.76, 0.43); *p* = 0.0840	−1.17 (−3.58, 1.24); *p* = 0.3397	−2.37 (−3.86, −0.88); *p* = 0.0019	−0.05 (−3.10, 3.01); *p* = 0.9766	3.37 (−0.47, 7.20); *p* = 0.0850	1.90 (−2.23, 6.04); *p* = 0.3654
Estimated Mean change (95% CI) from baseline to the last CHW visit in each group	Low: −1.41 (−2.78, −0.05) High: −3.75 (−5.50, −1.99)	Low: −1.80 (−3.74, 0.13) High: −4.97 (−8.92, −1.02)	Low: 0.32 (−1.14, 1.77) High: −0.86 (−3.46, 1.75)	Low: −1.21 (−2.61, 0.20) High: −3.57 (−4.85, −2.29)	Low: −3.74 (−5.53, −1.95) High: −3.78 (−6.94, −0.63)	Low: −0.34 (−3.02, 2.34) High: 3.03 (−1.90, 7.95)	Low: 2.62 (−1.17, 6.40) High: 4.52 (−0.44, 9.49)
**Mean difference in % change of weight**							
Exposure high vs. low	−0.79 (−1.46, −0.11); *p* = 0.0218	−1.19 (−2.93, 0.55); *p* = 0.1787	0.62 (−0.28, 1.51); *p* = 0.1780	−2.30 (−3.13, −1.46); *p* < 0.0001	−0.43 (−2.12, 1.27); *p* = 0.6200	−1.50 (−3.93, 0.94); *p* = 0.2266	−0.68 (−2.43, 1.07); *p* = 0.4452
Estimated Mean change (95% CI) from baseline to the last CHW visit in each group	Low: 0.22 (−0.34, 0.77) High: −0.57 (−1.28, 0.14)	Low: −1.55 (−2.48, −0.62) High: −2.74 (−4.66, −0.81)	Low: −1.14 (−1.68, −0.60) High: −0.52 (−1.49, 0.45)	Low: −2.29 (−3.06, −1.52) High: −4.59 (−5.31, −3.86)	Low: −1.62 (−2.62, −0.63) High: −2.05 (−3.80, −0.30)	Low: −1.84 (−3.53, −0.14) High: −3.33 (−6.47, −0.20)	Low: −2.06 (−3.66, −0.45) High: −2.74 (−4.84, −0.64)
**Change from HTN/elevated stage to normal BP stage**							
	**City A** **(***n*** = 784)**	**Town A** **(***n*** = 317)**	**Town B** **(***n*** = 404)**	**Rural A** **(***n*** = 445)**	**Rural B** **(***n*** = 306)**	**Rural C** **(***n*** = 294)**	**Rural D** **(***n*** = 442)**
Adjusted Odds Ratio and 95% confidence interval with *p*-value							
Exposure high vs. low	1.199 (0.790, 1.819); *p* = 0.3949	1.706 (0.531, 5.482); *p* = 0.3696	1.157 (0.576, 2.324); *p* = 0.6813	0.934 (0.518, 1.685); *p* = 0.8210	0.781 (0.366, 1.667); *p* = 0.5233	1.611 (0.405, 6.406); *p* = 0.4982	0.552 (0.091,3.334); *p* = 0.5173^*^
**Weight loss > 5% among the participants who had weight loss**							
	**City A** **(***n*** = 536)**	**Town A** **(***n*** = 292)**	**Town B** **(***n*** = 521)**	**Rural A** **(***n*** = 484)**	**Rural B** **(***n*** = 286)**	**Rural C** **(***n*** = 256)**	**Rural D** **(***n*** = 361)**
Adjusted Odds Ratio and 95% confidence interval with *p*-value							
Exposure high vs. low	1.432 (0.813, 2.521); *p* = 0.2141	3.356 (1.296, 8.693); *p* = 0.0126	6.017 (2.550, 14.196); *p* < 0.0001	2.404 (1.342, 4.307); *p* = 0.0032	0.653 (0.200, 2.130); *p* = 0.4795	1.461 (0.314, 6.789); *p* = 0.6289	0.368 (0.073, 1.855); *p* = 0.2256
**Change from obese to overweight/normal (pre-obese) BMI**							
	**City A** **(***n*** = 728)**	**Town A** **(***n*** = 269)**	**Town B** **(***n*** = 437)**	**Rural A** **(***n*** = 346)**	**Rural B** **(***n*** = 255)**	**Rural C** **(***n*** = 237)**	**Rural D** **(***n*** = 324)**
Adjusted Odds Ratio and 95% confidence interval with *p*-value							
Exposure high vs. low	1.120 (0.470, 2.666); *p* = 0.7985	5.112 (1.280, 20.427); *p* = 0.0210	2.355 (0.827, 6.706); *p* = 0.1086	0.981 (0.449, 2.146); *p* = 0.9623	1.917 (0.552, 6.661); *p* = 0.3057	6.690 (0.982, 45.584); *p* = 0.0522	1.260 (0.149, 10.654); *p* = 0.8318

*Linear regression models were performed for the mean difference in the % change of SBP, DBP, and Weight in each location. Logistic regression models were performed on all individuals who had Hypertensive or Elevated Stage of BP at baseline, who had weight loss comparing last visit to baseline, or those who were obese at baseline in each location. All estimates are based on multivariable models after controlling for number of program strategies received, duration of follow-up, baseline age, gender, insurance status, poverty status, and baseline comorbidity such as diabetes, hypertension and obesity*.

* *Estimate is based on a multivariate model not including poverty status as a controlling factor due to a major missingness in the factor*.

Participants' DBP levels were improved in both high and low exposure groups from City A, Town A, Rural A, and B. The high exposure group had more reduction in DBP in all locations except Rural C and D, but this finding was statistically significant only for the participants in City A, and Rural A.

Both the high and low exposure groups had weight loss except for low exposure group in City A. More weight loss was found in the high exposure group compared to the low exposure group in all locations except Town B (both high and low exposure group had weight loss, but more loss was found in low exposure group in Town B), and this finding was statistically significant for the participants in City A and Rural A (Table 6).

Among those who had hypertensive or elevated BP at baseline we found that high exposure group was more likely to recover to normal BP level from hypertensive or elevated BP than low exposure group for the participants in City A, Town A, B, and Rural C, but these findings were not statistically significant. Among the participants who had weight loss, we found that the high exposure group was more likely to achieve a minimum 5% weight loss compared to the low exposure group in all locations except Rural B and D, and this improvement found in Town A, B and Rural A was statistically significant. Among those who were obese at baseline, we found that TSSC program helped participants improve to either overweight or normal weight in all locations except Rural A. This finding was found statistically significant in Town A, and marginally significant in Rural C (Table 6).

Risk factor screening is a core component of the program. The goal is to identify individuals with previously unknown conditions such as hypertension, educate, and motivate them to address their condition through CHW-based motivational interviewing resulting in lifestyle changes, and refer them to healthcare services. Among 3,842 participants who believed themselves to have normal blood pressure (no self-reported, pre-program diagnosis of hypertension by a provider) prior to the study, 65.6% (*n* = 2,517 out of 3,842) were identified through the TSSC program of their elevated blood pressure status. Furthermore, 41.1% of those with newly discovered abnormal blood pressure at baseline improved to a lower blood pressure risk category or to normal blood pressure by the last CHW visit (*n* = 1,575 out of 3,828 with follow-up blood pressure readings). When we limit our analysis to only individuals with hypertension (BP > 140/90) at baseline, the proportion of those who improved their blood pressure to a lower risk blood pressure category by the last CHW visit among those who were newly diagnosed was not significantly different from those who knew about their hypertension status before the start of the study (70.13 vs. 67.24%; *p* = 0.1428).

### Adoption

During the first two thirds of the study period (January 2014 to December 2017), nine locations were offered the program and all nine adopted it in one county (100% adoption rate). A total of 18 municipalities are in this county. Beginning August 2017, more locations were approached. One municipality (Rural F) was approached and adopted in the initial county. Three out of four precincts in a second county were contacted, and two implemented the program. One county precinct chose not to participate because their existing programming for health met the needs of their area. This precinct did not differ on sociodemographic variables such as racial composition or poverty rate as compared to the adopting precincts (34). All locations that adopted were provided funds to cover the costs of the one CHW per location and some program funds to support items like computer equipment and weight and blood pressure monitoring equipment. The overall adoption rate across both counties (12 adopted/13 approached) was 92.3%.

### Implementation

Seven of the 12 locations implemented with fidelity during this time period (58.3%) and had a sample size sufficient (*n* ≥ 35 in both the low and high exposure group with documented implementation of the TSSC curriculum) to be included in the location-specific effectiveness analysis (reported above). A map of the locations is provided in Figure 1. The remaining five locations were determined to lack fidelity because personnel issues (City B and Rural E), delayed implementation from administrative setbacks, or lack of certified CHWs employed (Town C, D, Rural F) negatively impacted the program's home visit implementation and thus, insufficient numbers of participants received the required curriculum.

### Maintenance

Eleven of the 12 locations maintained the program (91.6%) through November 2019. The one location (Rural E) that discontinued the program did so due to personnel issues.

## Discussion

There is growing scientific evidence of the contribution CWCs make in the management of chronic diseases and related behaviors (36, 50–55), including among Latino populations (15, 17, 56). Our study provides evidence of public health impact of a CWC intervention in the areas of hypertension and obesity outcomes among Latino populations. Uniquely this study used the RE-AIM Framework to examine the Reach, Effectiveness, Adoption, Implementation and Maintenance of the Tu Salud ¡Sí Cuenta! Community-wide Campaign across multiple locations in a scaled-up implementation effort over a timeframe of nearly 6 years (50).

### Reach

This study found that the CWC intervention element of providing community health worker health education reached over 15,000 people or just over 2% of the total population across the locations. Additionally, the intervention effectively enrolled the population prioritized for these programming services–in this case low-income Latinos. This is a key component of “Reach” from the RE-AIM Framework which is not regularly or accurately reported in Community based interventions, and when it is, there is evidence that the prioritized groups are over or under-represented (50, 57). For example, in one worksite wellness intervention, employees from higher income households, with higher education levels and health literacy proficiency were significantly more likely to participate in the program (*p* < 0.01) (58). In another community health promotion intervention for African American and Latina women, they found that African Americans were more likely to not meet eligibility criteria and that the Latina women were more likely to drop out (57). Moreover, there were no significant differences by recruitment method or city, but overall participants were overrepresented by higher educated, wealthier, and older women (50, 57). Future research on recruitment strategies to address disparities and equity would be appropriate (50, 57). This TSSC study offers a successful example of enrollment strategies reaching the intended population. The TSSC program will need to expand the percentage of the participants who are exposed to the intervention components at a high level in future implementation efforts.

### Effectiveness

The TSSC study found significant improvement in blood pressure and weight status across the locations, with those in the high exposure group being associated with a generally greater improved blood pressure and weight status than those in the low exposure group, but with both groups showing improvement. We controlled for factors where there was an imbalance at baseline including age, insurance status and federal poverty level in order to isolate the program effects through our multivariable analyses. The study population was dominantly low income, uninsured, middle-aged Latinas, and therefore our findings provide unique insight into controlling chronic disease in this population. To our knowledge this study is the first to report hypertension and obesity-related outcomes associated with exposure to a CWC intervention among a purely Latino population. Other community-based interventions, however, have achieved hypertension improvement (59) and weight loss (60–65) including Latino populations. Finally, this study's CWC model was driven by CHW outreach, which has been associated with improved behavioral and health outcomes among Latinos (17, 18).

Analysis examining change in national blood pressure categories showed an association that approximately half of each exposure group's individuals with baseline hypertension reduced risk to pre-hypertensive or normal blood pressure categories. It may be that blood pressure changes are more sensitive to the introduction of CWC strategies and CHW home-visits compared to the program's weight loss outcomes. It may also suggest that the CHW risk factor screening and referrals provided motivation and support resulting in healthcare intervention for hypertension. Other community-based studies have found strong improvements in hypertension control (66–69). One was a multi-component community intervention including lay health workers studied as part of a large randomized control trial and found long term improvements in hypertension in South Asia (69). Hypertension and obesity are often linked conditions, both of which intrinsically cause negative health ramifications but synergistically can lead to even more debilitating adverse health impacts that are difficult to holistically treat and address (60). Moreover, improved hypertension in the majority of our study sample not only has the unilateral impact of improving cardiovascular and cerebrovascular-related morbidity, but it induces physiologic changes such as reductions in insulin resistance, enhanced sodium retention, and changes in natriuretic peptide, that enhances weight loss compared to patients who are seeking weight control with uncontrolled hypertension status (60–71).

In line with the previous literature (5, 7, 46) a 5% weight loss criterion was set as the goal for our population as the magnitude of weight loss observed was modest overall. Indeed, out of over 5,000 individuals in our analysis, ~100 reduced their weight enough to move into a lower BMI category. Even those remaining in the same BMI category, achieving any form of weight loss of 5% or greater will yield long-term health benefits in terms of glycemic control, blood pressure reduction, and all-cause mortality (5, 7, 46) which is important for the long-term implications of this health promotion CWC for this vulnerable population.

Our results indicate females were more likely than males to have significantly greater improvement in hypertension and weight. Other community-based studies have found this as well (72–74). This may have been the case because the strategies for exercise and nutrition implemented by the TSSC program were more appealing to women rather than men, or available during times when females could engage more easily. The program has expanded offerings geared toward men, but further efforts may be needed to ensure both men and women, equally, benefit from participation in the program.

We found that having insurance was related to less improvement in SBP and DBP. This may partially be explained by the fact that those with insurance may already have access to medication for their hypertension and have somewhat better control of the condition and, therefore, have less movement toward improvement. Other studies have found that having health insurance is related to better hypertension control (75). In the region in which this study occurred, insurance could be a confounding factor in relation to the TSSC effect on each outcome since having insurance likely equates to individuals having access to care and medications for hypertension and other non-communicable diseases that can also influence the level of blood pressure decrease. Having insurance can also be related to having more stable employment, higher socioeconomic status, and legal residency/citizenship status and therefore it is not surprising to find significant differences by insurance status and ability to engage in the program (76).

During the 6-year timeframe of the study, we expanded the implementation of the program to 12 locations. Of those, seven reached a minimum threshold of implementation where at least 35 participants had documented high level exposure to the program's curriculum delivered through home-visits allowing us to examine low exposure vs. high exposure by location. A clear pattern resulted from this analysis. Nearly every location found improved outcomes in the high exposure group vs. the low exposure group across SBP, DBP, and weight loss. Two cities (City A, Rural A) consistently achieved statistically significant differences by exposure group on these outcomes. Both of these cities had >30% of their enrolled population in the high exposure group. The other locations had <15% of their enrolled population in the high exposure group (between 6.7 and 13%) suggesting that our location specific analysis was unable to reach the statistically significant level of difference between the high and low exposure group in these later locations. Therefore, as the program continues to mature and more sites are able to ensure that participants are receiving the curriculum through the multiple visits and are exposed to the other components of the intervention so as to achieve a high level of exposure to the program, our ability to detect statistically significant results may improve.

### Adoption

The CWC was readily adopted by all but one location. A factor influencing the adoption of the program was that each location was assured that the personnel and activity costs would be covered by an outside funding source. Therefore, the decision for city officials to adopt the program rested in their commitment and interest in health, and the balancing of other competing programs in promoting health. Past research has shown that the main reason interventions are not adopted is their lack of relevance (77–79). Other factors influencing adoption include characteristics of the innovation and traits of the target audience (80). Research has also found that information about staffing, intervention delivery, and organizational burden are rarely discussed but central to adoption decisions (81, 82). However, in this study, because we had years of experience implementing the CWC intervention in one location, prior to the scaling up of the program, we were able to answer questions about staffing, intervention delivery, organizational burden, collaboration with other sites, and benefits and barriers for government staff and community members.

### Implementation

In general, the implementation of the program components across locations was directed by the university to ensure fidelity to the model. For example, the media for the program was produced by the university, but captured location-specific stories. Where implementation tended to falter was in finding qualified personnel who could deliver the home visits. Some locations struggled to find community health workers with experience or capacity to lead the program efforts in a culturally, and linguistically appropriate way, causing some delays in the program launch or low enrollment of participants in the home visits delivered by CHWs. Slightly over half the locations were considered to have implemented the program with fidelity, meaning that the expectation of a sufficient dose of monthly home visits using the TSSC curriculum was implemented to achieve a health outcome. Other research has found that fidelity to implementation included monitoring (1) program involvement of key community stakeholders, (2) personnel training, (3) monthly monitoring of the dosage of program components, (4) the adherence to program component protocol at each site, and (5) recording attendance logs and meeting minutes (83). In our program implementation, we conducted CHW monthly training sessions to ensure programming consistency and time for troubleshooting. We also conducted annual implementation evaluations with location-specific policy leaders, CHWs, and community leaders. Future analysis should explore these data by location.

### Maintenance

This study found that all but one location maintained implementation during the nearly 6-year time frame providing long-term opportunities for exposure to the program for participants. However, one of the original CWC implementation efforts occurred in Finland and found improved blood pressure outcomes over a sustained program across 40 years (22, 23).

## Limitations

There are limitations to the study, including that the study lacked a no-treatment control group. However, as this study was focused on real-world, scaled up implementation of a program, and past research on the model has provided more stringent study design options (17, 19, 84). We believe that the results of this implementation study do contribute uniquely to the literature. Another limitation is how BP variability in the protocol can influence results, especially related to differential positioning of BP readings, calibration of BP instruments, and temporal relation of measurements in regard to if the BP is taken immediately after a class activity as this can lower BP readings and lead to over-pronounced program effects if compared to baseline BP readings done before activity.

In order to control for many of these issues, staff met monthly to share best practices and completed a yearly training on all blood pressure protocols and measurement procedures. Standard protocols were in place for CHWs to follow when participants had blood pressure readings in the hypertensive crisis range. The protocol triggered an urgent care referral by CHWs.

A significant limitation of the study was that during the study period, the home-visit protocols did not ask participants about concurrent use of medications, such as anti-hypertensives. This void in our data limit our results, particularly because we have little information about if such medications were started between the initial and the follow-up visit. What data we do have was available for a limited number of participants (*n* = 456). Among those who were referred to a primary care provider, 94.5% were hypertensive (stage 1 or 2) or in hypertensive crisis (*n* = 431 out of 456), and about half of them were not diagnosed with hypertension prior to the study (*n* = 228). This is suggestive that about 50% of our participants could have begun antihypertensive medications if they were able to access primary care providers during the timeframe of their CHW study visits. As stated, the median follow-up time for a participant was 3.23 months in the low exposure group and 6.53 months in the high exposure group. Primary care in our region during this time, particularly for low-income, uninsured populations was experiencing a 12–18 month wait period. Therefore, we estimate far <50% of the participants who newly found about their abnormal blood pressure were able to see a provider and begin prescribed medication during the median follow-up timeframe of the study. Future iterations of the program should measure completed healthcare referrals, and medication usage specifically. Medication usage data would also be helpful to control for the confounding effects medication usage can have on blood pressure reduction and weight loss.

This perhaps highlights the holistic and integrated nature of this CWC-based intervention. It is not necessarily that CHW home visits itself cause blood pressure reduction, but the entire multimodal components of the TSSC intervention- of which the CHW home visits are a core component of- precipitate into tangible blood pressure reduction. Specifically, this includes: one, CHW-based home visits where vital signs are measured causing individuals in the local community to become aware of their hypertension status; two, resultant CHW-led motivational interviewing-based conversations to counsel and motivate participants to seek treatment and engage in lifestyle modifications; and three, the TSSC intervention providing built environmental opportunities for participants to engage in these lifestyle modifications including physical activity and healthy cooking classes, social support, messaging, and social services referral. The culmination of the different facets of this entire CWC program is likely necessary to derive blood pressure reduction in this at-risk population. Our analysis quantified program exposure based on CHW home visits as that aspect of the program was most readily and meticulously documented during this dissemination and implementation project. Program improvements for the future will include being able to accurately document the number of participants who are successful in seeking external medical care and to determine blood pressure changes in individuals with no medication usage to quantify the isolated effect of the program on blood pressure reduction.

In regards to overweight measures, it is possible that individuals' weight fluctuated far more than what the CHWs measured on the participant. Staff did not review participants' daily or weekly logs of their weight, although logging was a practice that was encouraged, therefore, the only measures we have of weight were staff measures at designated time points. The timeframe of the study, particularly by location, did not allow for a longer-term assessment of the longitudinal impacts of this CWC on the studied population. Finally, there is a possibility of self-selection bias among the studied population. The high exposure group had significantly more individuals who had hypertension and overweight/obese status compared to the low exposure group. It is probable that individuals who knew they had more serious non-communicable disease status (high blood pressure and overweight/obesity) were more motivated and inclined to engage more in the programming for its expected health benefits than those with less severe medical comorbidities. It is also possible that CHWs are more likely to focus on following-up on participants with abnormal blood pressure, BMI, and comorbidities due to the follow-up protocol which prioritized more rapid follow-up for participants with blood pressure measures in the hypertensive range. Lastly, during the period of this study, the availability of weight-loss medication was not as readily present as today and thus there is less concern about the confounding effects of medication usage contributing to observed weight changes seen over the course of the study.

## Conclusion

This study utilized the RE-AIM framework (29, 35) to evaluate the public health impact of a scaled-up CWC and its association with reduced blood pressure and weight among a medically underserved population of low-income Latinos along the U.S.-Mexico border region, who have a disproportionately high prevalence of hypertension and obesity (3). The study showed effectiveness; as TSSC program participants were exposed to a high level of the program including the CHW follow-up visits, the participants also reported significant decreases to blood pressure and BMI, with this impact moderated and magnified by increased program exposure. Furthermore, findings indicate program participants are more likely to lose 5% of weight or more with increased program exposure, highlighting the importance of implementing a variety of strategies to achieve a decrease in diabetes incidence (5). This, thus, emphasizes the universality of the CWC approach in its ability to promote the adoption of healthier behaviors with positive blood pressure and BMI outcomes in this health disparate region. Future studies may also wish to evaluate the effect of comorbidities on outcomes, and should document medication adherence related to outcomes. The study also documented high adoption rates, moderate implementation fidelity, and high program maintenance across a wide array of communities ranging from rural environments to urban neighborhoods. Evidence-based programs focusing on the application of health behavior promotion in Latino communities are often limited in scope, with little published literature emphasis on the sustainability of the intervention (50). This study adds to the literature an exemplification of the real-world reach, effectiveness, adoption, implementation, and maintenance of a regional scaled-up CWC that provides promise for augmenting the population health trajectory of an entire region's medically underserved Latino population.

## Data Availability Statement

The raw data supporting the conclusions of this article will be made available by the authors, without undue reservation.

## Ethics Statement

The studies involving human participants were reviewed and approved by Committee for the Protection of Human Subjects the University of Texas Health Science Center at Houston. Written informed consent for participation was not required for this study in accordance with the national legislation and the institutional requirements.

## Author Contributions

BR, LM-B, ML, and PY conceptualized, wrote, and edited paper. ML, SP, and TX performed statistical analysis. AD and AO-D wrote sections of the manuscript and managed data collection. All authors contributed to manuscript revisions, and read and approved the submitted version.

## Funding

This work was funded by the project came from the Texas Department of Health and Human Services, SNAP ED contract #529-17-0046-00004. The research was partially supported by the Center for Clinical and Translational Sciences funded by NIH NCATS UL1 TR003167. The funders did not have a role in the research presented in this study.

## Conflict of Interest

The authors declare that the research was conducted in the absence of any commercial or financial relationships that could be construed as a potential conflict of interest.

## Publisher's Note

All claims expressed in this article are solely those of the authors and do not necessarily represent those of their affiliated organizations, or those of the publisher, the editors and the reviewers. Any product that may be evaluated in this article, or claim that may be made by its manufacturer, is not guaranteed or endorsed by the publisher.
